# Regulating scientific and technological uncertainty: The precautionary principle in the context of human genomics and AI

**DOI:** 10.17159/sajs.2023/15037

**Published:** 2023-05-30

**Authors:** Marietjie Botes

**Affiliations:** 1 SnT Interdisciplinary Centre for Security, Reliability, and Trust, University of Luxembourg, Luxembourg, Luxembourg

**Keywords:** precautionary principle, risk-based approach, human genomics, AI, regulation

## Abstract

Considered in isolation, the ethical and societal challenges posed by genomics and artificial intelligence (AI) are profound and include issues relating to autonomy, privacy, equality, bias, discrimination, and the abuse of power, amongst others. When these two technologies are combined, the ethical, legal and societal issues increase substantially, become much more complex, and can be scaled enormously, which increases the impact. Adding to these complexities, both genomics and AI-enabled technologies are rife with scientific and technological uncertainties, which makes the regulation of these technologies not only challenging in itself, but also creates legal uncertainties. In science, the precautionary principle has been used globally to govern uncertainty, with the specific aim to prevent irreversible harm to human beings. The regulation of uncertainties in AI-enabled technologies is based on risk as set out in the AI Regulation that was recently proposed by the European Commission. However, when genomics and artificial intelligence are combined, not only do uncertainties double, but the current regulation of such uncertainties towards the safe use thereof for humans seems contradictory, considering the different approaches followed by science and technology in this regard. In this article, I explore the regulation of both scientific and technological uncertainties and argue that the application of the precautionary principle in the context of human genomics and AI seems to be the most effective way to regulate the uncertainties brought about by the combination of these two technologies.

## Introduction

Human genomics has the potential to provide an efficient and cost-effective means of preventing, diagnosing, and treating major diseases that burden populations and enables the tailoring of medicine to the specific needs of individuals. However, the exact impact of this rapidly evolving scientific field on diagnostic and therapeutic health services, and how it will affect societies, are still largely uncertain and subject to ongoing research. Since the completion of the draft human genome sequence more than 20 years ago, an extraordinary amount of genomic data has been generated, which will only increase in volume and complexity alongside the increase in genomic sequencing and related biological techniques. These circumstances force genomics researchers to turn to artificial intelligence (AI) and related machine learning (ML) based computational tools to help them extract, interpret, and analyse information from these valuable data sets into formats that can be used and translated into meaningful outcomes and effective treatments. Similar to human genomics, computer scientists are also continuously developing new techniques and technologies in their field of AI and ML, making it very dynamic, but also very complex and uncertain, which seems to be one of the most common and difficult problems to solve in AI-enabled technologies.^1^

Regardless of the fact that the combination of genomics and AI/ML has only started fairly recently, some of the medical breakthroughs it envisions include

examining people’s faces with facial analysis AI programs to accurately identify genetic disorders; using ML techniques to identify the primary kind of cancer from a liquid biopsy; predicting how a certain kind of cancer will progress in a patient; identifying disease-causing genomic variants compared to benign variants using machine learning; and using deep learning to improve the function of gene editing tools such as CRISPR.^2^

But despite the positive changes that these technologies promise, one cannot ignore that they are founded on rapidly developing and ever-evolving genomics *and* AI/ML technologies – fields that are both rife with scientific and technological uncertainties, and which uncertainty is merely exacerbated by their combined use, which in turn creates regulatory uncertainties.

Some of the most pressing ethical, legal, and societal issues associated with the combination of human genomics and AI/ML were presented by Farmer^3^ during the Global Alliance for Genomics and Health’s (GA4GH) 10th Plenary Meeting in September 2022 and are summarised in Table 1. Although AI-powered genomics enhances the collection of data and the accuracy of genomic analysis, it still presents problems relating to missing data, bias, privacy, consent, and genetic discrimination in general. Due to its speed and ability to scale, AI has not only exacerbated these problems, but also added new ones such as interpretability, explainability, accountability, and enabling the ease with which more sensitive inferences can be drawn from genomic data – all whilst life sciences and big tech operates with critically different business models, incentives, cultures, and approaches to ethics.

The aim of this paper is not to discuss the various ethical, legal, and social implications (ELSI) and related issues in detail, but to compare the precautionary principle that is widely used in genomic research with the risk-based approach embedded in proposed AI legislation, and to analyse the appropriate regulatory approach to govern scientific and technological uncertainties that will support scientific and technological innovation, without compromising the safety of people. Reference to the numerous ELSI with regard to the combined use of genomics and AI/ML serves to indicate the complexity of both of these large and emerging research fields, and how their inevitable combination adds to such complexity and uncertainty in their regulation.

### Challenges posed by secondary findings in genomics

Genomic research often reveals ‘unsolicited’ or ‘incidental’ findings that may be important to the health, treatment, or future health of participants. While it is widely accepted that researchers have a moral obligation to disclose and report secondary findings to participants if there is effective treatment available for the specific health condition with an immediate onset, researchers are less widely considered to have a moral obligation to actively search for health-related findings, especially if it falls outside the scope of the research project.^4^ Koplin et al.^4^ argue that the only reason that genomic researchers are currently not morally obligated to actively search for secondary findings is because the present costs involved in doing so still far outweigh likely benefits to the participants. However, by combining genomic research with AI/ML, researchers may soon acquire a moral obligation to actively search for secondary findings in the near future when the process of searching for such findings becomes more cost-effective, and serious harm to participants can actually be prevented through rapid improvements of technologies and treatments. But to what extent the benefits to participants must outweigh the costs associated with looking for secondary findings, to determine the moral duty of genomic researchers, is and may remain very uncertain. In an effort to provide guidance in this context, the American College of Medical Genetics published a list of medically actionable secondary findings that researchers must look for and report when doing clinical genome sequencing.^5^ But being non-binding recommendations, only some researchers strictly followed these suggestions, whilst others were reluctant to do so due to their concerns with the medical reality that only a small percentage of genetic variants associated with disease would actually result in participants manifesting with disease.^6^ Despite an updated list of medically actionable findings to return secondary findings, published by the American College of Medical Genetics, there is still no consensus among researchers, clinicians, and bioethicists about when, what, and how secondary findings must be sought or returned when found.^7^ In addition, a growing number of studies that investigate the preferences of the general public, patients, and research participants in this regard, including the impact on these groups of people upon receiving secondary findings, indicates that policies about the returning of secondary findings will be strongly influenced by increased public understanding of genomics and their subsequent preferences, alongside the views of experts.^8^

Further arguments on whether to report secondary findings trigger numerous ethical questions relating to autonomy, non-maleficence, and beneficence, – principles which are often contradictory to one another and in themselves inadequate to justify a fair and reasonable solution. In this regard, Saelaert et al.^9^ argue that the mandatory reporting of actionable secondary findings could even be interpreted as a “technological, soft paternalism” when participants’ choices or access to their personal information are restricted by scientists, but may be ethically acceptable if the motives behind such restrictions are valid and the beneficial outcome for the participant is very likely. Subsequently, a patient’s inability to make informed decisions relating to their future treatment, normative rationality, the efficacy of outcomes that may be beneficial to the patient, and how that beneficence should be determined, must be considered critically.

Even the seemingly simple act of recontacting participants after genetic and genomic research results have been reinterpreted is a complex issue involving a network of clinical and research laboratories, clinicians, and researchers across specialties. At present, the recontacting of participants necessitated by research findings occurs on an ad-hoc basis which may lead to information being provided only to those participants who can be easily located, or only in so far as research funding allows this to occur. To provide much needed guidance in this regard, the American Society of Human Genetics issued a position statement containing recommendations on how to operationalise the recontacting of participants, including when and how this should be done.^10^ Although these recommendations provide a set of principles researchers can use when they anticipate situations in which the return of study findings and the recontacting of participants may become appropriate, the operationalisation of these principles is still subject to institutional ethical review and the purview of advisory boards with regard to the practical implementation thereof.^11^ In addition, these recommendations were issued in the midst of an evolving genomic and technological landscape with rapid changes occurring in IT, including AI/ML, which in turn will have significant influences on society’s beliefs, values and approach to the implementation of these recommendations. Accordingly, recommendations and policies in this regard will have to be updated on a regular basis to keep pace with scientific and technological developments. It is in this context that the precautionary principle in genomic research finds its application to ensure the equitable and effective delivery of high-quality research results, including to those who participate in research.

For many of the above reasons, technological pessimists who fear the appearance of so-called ‘sorcerer’s apprentices’, advocate for stringent regulation of genomic activities; in contrast, technological optimists seem to have complete faith in the scientific progress and oppose regulation based on their argument that regulation acts only to stifle scientific progress. The precautionary principle poses a useful method of thinking to appease both the concerns of technological pessimists, whilst still allowing enough regulatory room for scientific innovation to thrive, specifically in circumstances in which genomic research activities and/or the application of cell and gene therapies poses uncertainty and potentially both success and risk. But, to consider the place and function of the precautionary principle in the combination of genomic science and AI/ML technologies, the extent and consequences of involving AI and ML in genomics must also be considered.

### Challenges posed by AI/ML based computational technologies

Despite the potential that AI/ML holds for genomics and health care in general, some of the ethical issues associated with AI/ML, highlighted in a 2021 study by Stahl, specifically those most relevant to genomics, include

cost to innovation, harm to physical integrity, lack of access to public services, lack of trust, security problems, lack of quality data, power asymmetries, negative impact on health, problems of integrity, lack of accuracy of data, lack of privacy, lack of transparency, potential for military use, lack of informed consent, bias and discrimination, unfairness, unequal power relations, misuse of personal data, potential for criminal and malicious use, loss of freedom and individual autonomy, contested ownership of data, reduction of human contact, problems of control and use of data and systems, lack of accuracy of predictive recommendations, lack of accuracy of non-individual recommendations, violation of fundamental human rights of end users, unintended, unforeseeable adverse impacts, prioritisation of the ‘wrong’ problems, negative impact on vulnerable groups, lack of accountability and liability, loss of human decision-making, and lack of access to and freedom of information.^12^

Stahl’s^12^ long list of ethical concerns not only shows us the uncertainty that AI/ML technologies bring along, but also cautions us not to reproduce, legitimise, and aggravate these concerns by unquestioningly implementing AI/ML in genomics.

In an effort to regulate some of these concerns, the European Union (EU) published a draft regulation for Artificial Intelligence (AI Regulation) on 21 April 2021, but none of the practical summaries, comments, or presentations contained in this draft deals with the fundamental question of how to regulate the above concerns and uncertainties brought about by AI/ML. Being fully aware of the uncertainties and risks that AI poses, the EU opted to introduce a risk-based approach for the regulation of risks associated with AI systems, based on three tiers: (1) unacceptable risk – which simply bans the use of any AI systems posing unacceptable risk; (2) high risk – which subjects high-risk AI systems to extensive technical, monitoring, compliance and transparency obligations; and (3) low risk – systems which are encouraged to self-regulate by implementing codes of conduct.^13^ Once the highest compliance risks to an organisation have been identified and the organisation manages to successfully reduce the identified risks with the prescribed compliance methods and tools, the AI system risk level can then be reduced to a lower one. From the perspective of using such a risk-based approach to protect data, the Article 29 Data Protection Working Party already stated in 2014 that the risk-based approach must span beyond a narrow “harm-based-approach” that only focuses on the prevention of damages, and that it should also take into account

every potential as well as actual adverse effect, assessed on a very wide scale ranging from an impact on the person concerned by the processing in question to a general societal impact (e.g. loss of social trust).^14^

The draft AI Regulation defines AI by referring to software systems that generate outputs for human-defined objectives (which explains its application in the field of genomics) as:

…software that is developed with one or more of the techniques and approaches listed in Annex I and can, for a given set of human-defined objectives, generate outputs such as content, predictions, recommendations, or decisions influencing the environments they interact with.^15^

In Annex I of the draft Regulation, almost every technique currently known that relates to ML approaches, logic- and knowledge-based approaches, and statistical approaches is listed.^15^ This list was clearly intended to encapsulate a very broad spectrum of AI systems, but whilst doing so also includes “very unspecified objects”^16^. Legally speaking, this approach to regulate a very broad range of unspecified technologies with uncertain uses, outcomes, and consequences is extremely undesirable.

It seems that the European Commission tried to regulate risky *techniques* in general, instead of focusing on AI as a technology that employs some of these risky techniques. The effect thereof is that simple existing technologies such as the pocket calculator may be considered as AI in terms of the definition of AI and the techniques listed in Annex I. This situation will inevitably subject most, if not all, technologies using one or more of the techniques mentioned in the draft AI Regulation to stringent compliance regulations, and thereby possibly slow down the uptake, use, and implementation of technologies that do not pose serious technological risks. Rather, the goal of any AI act or regulation, as envisioned by the Article 29 Data Protection Working Party in 2014, should be to protect people against harmful inventions that threaten our fundamental rights, whilst avoid dampening innovation. Ironically, this is exactly the goal of the precautionary principle, but with one big difference: the precautionary principle is not codified in legislation.

The regulation of risks arising from uncertainties, especially those brought about by the combination of genomics and AI/ML, requires an approach from different perspectives because of the many unanswered ethical questions that remain, as discussed above. Accordingly, I will argue that an innovative technology should not only be considered and legislated with regard to its capabilities or its need to respect certain ethical principles, it must also be considered in light of the precautionary principle, having regard to possible irreversible damages, bias and inequity, privacy issues, and discrimination it may cause.

### The precautionary principle

Legislation and associated regulations are not ideal tools that can provide immediate protection against pressing scientific or technological harms. These require a much longer and protracted process from drafting a bill to final enactment. In contrast to this process, and although no universally accepted definition of the precautionary principle exists, the precautionary principle is considered to enable decision-makers to adopt precautionary measures promptly when scientific evidence about an environmental or human health hazard is uncertain and the risks to human life and society are high.^17^

The precautionary principle has its origins in international environmental protection^18^, and was incorporated into almost all international treaties on environmental protection since the 1990s to the extent that France even incorporated this principle into its Constitution in 2005^19^, with Sweden, Belgium, the Netherlands and Australia formally incorporating it into their national environmental policies. This principle then became widely applied by states, in accordance with their national capabilities and where threats of “serious or irreversible damage, *lack of full scientific [and technological] certainty* shall not be used as a reason for postponing cost-effective measures to prevent environmental degradation” (my addition and emphasis).^20^ The precautionary approach is thus a broad epistemological, philosophical, and legal approach to innovations that pose a potential for causing harm when extensive scientific knowledge, and I will add technological knowledge, on the matter is lacking. It emphasises caution, pausing, and review before leaping into new innovations that may prove disastrous.

Whilst there is still no global consensus on the legal status of the precautionary principle in the context of international law, the European Union Court of Justice explicitly stated that:

the precautionary principle can be defined as a general principle of Community law requiring the competent authorities to take appropriate measures to prevent specific potential risks to public health, safety and the environment, by giving precedence to the requirements related to the protection of those interests over economic interests.^21^

And the European Commission is of the opinion that “this principle has been progressively consolidated in international environmental law, and so it has since become a full-fledged and general principle of international law”^17^.

Even though South Africa is a signatory to the Rio Declaration which imported the precautionary principle into South Africa’s policy frameworks, the precautionary principle has had limited national practical application, and I agree with Glazewski and Plit^22^ that the active implementation of this principle should be given serious consideration, especially considering South Africa’s national development agenda. South Africa’s National Development Plan 2030 states that “science and technology are fundamentally altering the way people live, connect, communicate and transact, with profound effects on economic growth and development” and the application of the precautionary principle will be fundamental to the furthering of “technological and scientific revolutions which underpin economic advances, improvements in health systems, education and infrastructure”^23^. In addition, considering that the South African government considers Europe to “continue to be South Africa’s biggest trading partner for some years to come”^24^, and Europe’s stance on the status of the precautionary principle as discussed above, it is advisable that this principle be implemented into scientific and technological developments sooner rather than later.

The scope and extent of the implementation of this principle will depend on prevailing social and political values and could be developed further in case law resulting from legal action, which makes this principle an ideal tool to dynamically regulate the uncertainties of emerging sciences and technologies in line with socio-political developments without amounting to legal uncertainty. A key variable in this regard is the degree of scientific or technological uncertainty that would likely mobilise authorities into action, having due regard to the severity and probability of the risks involved, the magnitude of the stakes, and the potential costs of action or inaction. However, I agree with Stirling^25^ that although a science-based risk assessment offers a powerful method to determine strict states of risk, it is not applicable under conditions of uncertainty, ambiguity and ignorance and such reductive methods, in the absence of a strict state of risk, may prove to be irrational, unscientific, and potentially misleading. From a regulatory perspective, the quantification of risk, or a definitive expert judgement on safety, is of immense value for purposes of creating concrete legislation; but, unfortunately, this has no rational scientific basis. It is also expected that robust legislation must address long-term issues for effective governance, where robustness is a result of the accuracy of assessment results, not of their professed precision, hence the seemingly impossible task to regulate scientific and technological uncertainties via legislation. Stirling continues to explain that the reason that so-called “sound scientific” procedures often yield contrasting pictures of risk, is based on the specific framing of the analysis of answers delivered in risk assessments, which in turn can dramatically influence the framing of science for policy. It is in this context that the value of the precautionary principle becomes clear.

The precautionary principle is not, and has never been claimed to be, a definitive decision-making tool, nor a detailed protocol that can be used to determine risks and uncertainties, but it does provide a general, yet dynamic, normative guide towards effective policymaking in times of uncertainty where the benefit of any doubt should be tilted towards the protection of human health, specifically in the case of AI/ML-enabled genomics. This means that the implementation of the precautionary principle requires a level of scientific and technological motivation and persuasion on the side of scientists and technologists with regard to the gathering of evidence. In these circumstances the value of the precautionary principle manifests in the fact that none of these issues can be dealt with in a strict scientific way. Instead, the precautionary principle demands the incorporation of a broader range of non-reductive methods that include a wide variety of methods to reveal the normative and contestable basis for decisions, to regulate scientific and technological uncertainties.

### Applying the precautionary principle

The main question is how to identify those cases that justify the application of the precautionary principle. In this regard, the 2005 report on the precautionary principle published by UNESCO’s World Commission on the Ethics of Scientific Knowledge and Technology, states that “when human activities may lead to morally unacceptable harm that is scientifically plausible but uncertain, actions shall be taken to avoid or diminish that harm”^26^. An answer clearly stated in the precautionary principle itself, and defined specifically as a response to lack of scientific certainty when there is a threat of serious or irreversible harm. Morally unacceptable harm, according to this report, is harm that threatens human life or health, is effectively irreversible, inequitable to future generations, or is imposed without consideration of the human rights of those affected, with the caveat that the plausibility of such harm must be based on scientific analysis and subject to review.

In the context of the combined use of AI/ML and genomics, the following framework for criteria may, for example, be used as a screening process to identify scientific and technological uncertainty, and most importantly, their impact on human life and health to decide whether to apply the precautionary principle:

- risks and harms posed to public and/or individual health and life and physical integrity
- degree and type of scientific and technological uncertainty
- presence or absence of morally acceptable harm
- impact of genomic secondary finding disclosure on fundamental rights of individuals and/or community
- reliability, accuracy, and bias of AI/ML-enabled genomic predictions and predictive recommendations
- benefit to individuals and/or society
- scientific and technological doubts about quality, accuracy, applicability, and transparency of data
- power asymmetries
- general violation of fundamental human rights
- novel, unintended, unforeseeable, unprecedented, or adverse impacts
- clear violation of risk-based concentration thresholds or standards
- scientifically and technological founded doubts on theory, model sufficiency, or applicability
- divergent individual or institutional perceptions of risk
- ethical, legal and social concerns, distributional issues or political mobilisation^27^

When none of the criteria in this framework is triggered, the AI/ML-enabled genomics application in question does not need the application of the precautionary principle, in which event the case will be subject to conventional risk assessment. Only when uncertainty is prevalent will it justify the initiation of a more precautionary approach. This hopefully shows how the precautionary principle does not present a blanket rejection of science, technology or even risk assessment, but rather triggers a careful consideration, measuring and approach towards the combination of genomics and AI/ML at different states of scientific and technological knowledge.

## Conclusion

In proposing the AI Regulation, the European Commission tried to regulate the technological uncertainty brought about by AI, by attempting to introduce some legal certainty via further risk-based thresholds, in addition to existing legal requirements. The risk-based approach, introduced by this AI Regulation, functions on the assumption that only those AI systems that pose a high or moderate risk to fundamental rights will fall within the scope of the risk categories as set out in the AI Regulation, meaning that only those AI systems need to comply with the requirements of the proposed AI Regulation. However, although the regulation of risks and harms are preferrable, this AI Regulation contains an overly broad definition of AI, as expanded upon in Annex I, which creates immense legal uncertainty as to what technologies actually fall within the ambit of the proposed regulation, over and above the technological uncertainties discussed above. Furthermore, if such a definition and expanded list of technologies is contained in a single AI act of any kind, any piece of legislation that tries to regulate any kind of software will find itself competing with the conditions set out in such an overarching act and possibly contain some contradictory clauses of its own. From a legal perspective, this scenario will only complicate the interpretation and application of legislation in scientific and technological fields which are already complex enough to govern due to rapid developments in these fields.

Thus, if we truly want to prepare ourselves for a functional, fair, reasonable, legal and ethical future in which the combination of genomic science and AI/ML serves human beings and contributes to the prospering of their existence, we should ensure that we capture scientific and technological techniques that may not be currently known, such as the combination of these technologies, instead of limiting ourselves exclusively to AI. Because absolute scientific and/or technological certainty, especially when combined, can never be achieved, the application of the precautionary principle in these circumstances can provide a dynamic framework that could help to achieve a better balance in genomics and AI/ML-based health outcomes and policies, whilst mitigating the difficulties presented by both scientific and technological uncertainty, before stringent regulations are enacted that may not be flexible enough to enable scientific and technological advancement. No single act, regulation, policy or guideline is enough to effectively protect fundamental rights and democracy and, most importantly, to avoid irreversible damage caused by the combined use of genomics and AI. The proposed AI Regulation, for example, does not deal with damages that may occur when applying AI to health care or in automated and opaque decisions, nor in the application of AI in the context of genomics. Hence the need to apply the precautionary principle in these circumstances to allow for the consideration of a vast array of governing instruments, ethical principles, and scientific and technological practicalities to allow for sustainable development in real time, whilst preserving the fundamental rights of both present and future generations.

### Recommendations

South Africa has no formal policy documents relating to AI, nor has it entered bills to parliament for the regulation of AI. Instead, AI is regulated under existing legal principles as and when applicable. Rather than reinventing the wheel, South Africa can learn from the EU’s attempts to regulate AI, and prevent many of the mistakes made in trying to govern AI per se. I therefore propose:

1. that any legislative effort in this regard must broaden the scope to rather regulate technologies, as opposed to limiting it to AI or ML exclusively, to allow for the long-term regulation of technologies, including those yet unknown;
2. to incorporate the precautionary principle into such legislation, much like the ethical principle of consent is now incorporated into legislation globally, to allow for the consideration of a broader spectrum of consequences when dealing with scientific and technological uncertainties; and
3. considering the combined use of genomics and AI/ML, any legislative effort should also include non-digital technologies that may pose a threat to our fundamental rights, such as certain bio-technologies – this should prevent regulations to be treated in a sectored way or without coordinated planning or with little technicality.

## Figures and Tables

**Table 1:** Ethical, legal, and social implications associated with human genomics and artificial intelligence / machine learning

Artificial intelligence / machine learning	Human genomics
Relies on mass data collection • creates incentives to undermine privacy • environmental impact of data storage	Genomics data privacy • the problem of secondary subjects • genome data are hard to anonymise • genomic data are particularly sensitive, and their value is hard to predict

Reliability Differential accuracy Bias	Reliability Differential accuracy Bias

	The genomic data double bind and ‘double vulnerability’

Explainability and interpretability	

Accountability for AI decision-making	

Subjection to AI decision-making	

Ownership of and benefit from AI and its outputs	Ownership of and benefit from genomic data • HeLa cells • Public think they own their genomic data

	Cost and opportunity cost • Question marks over the current value of genomic science • The value of investment in genomics compared to other interventions or research

## Significance:

The significance of this article rests in the criteria framework proposed for the determination of the applicability of the precautionary principle and lessons learnt from the European Union’s attempt to regulate artificial intelligence.
